# Evaluation of Recombinant Follicle-Stimulating Hormone (rFSH) Plus Recombinant Luteinizing Hormone (rLH) and rFSH Plus Human Menopausal Gonadotropin (HMG) Combination Therapies in Patients With Poor Ovarian Response Undergoing Ovarian Stimulation

**DOI:** 10.7759/cureus.104714

**Published:** 2026-03-05

**Authors:** Ebru Ersoy, Muhammed Hanifi Bademkiran, Mustafa Demir, Duygu Turk

**Affiliations:** 1 Obstetrics and Gynaecology, Faculty of Medicine, Gaziantep SANKO University, Gaziantep, TUR; 2 Obstetrics and Gynaecology, Faculty of Medicine, Gaziantep University, Gaziantep, TUR; 3 Obstetrics and Gynaecology, Gaziantep Islamic Science and Technology University, Gaziantep, TUR

**Keywords:** controlled ovarian stimulation, hmg, ivf, lh, poor ovarian response, rfsh+rlh

## Abstract

Objective: This study aimed to compare the clinical and laboratory outcomes of recombinant follicle-stimulating hormone (rFSH) plus recombinant luteinizing hormone (rLH) and rFSH plus human menopausal gonadotropin (HMG) combination therapies during controlled ovarian hyperstimulation (COH) in patients with poor ovarian response, with the live birth rate defined as the primary clinical endpoint.

Methods: This retrospective study included 90 patients who were diagnosed with poor ovarian response based on the Bologna criteria (which were also categorized as Poseidon Groups 3 and 4) and who met the inclusion requirements. Patients were divided into two groups based on the gonadotropin combination used: rFSH+rLH (n=49) and rFSH+HMG (n=41). Stimulation parameters, number of oocytes retrieved, embryo quality, and live birth rates were analyzed and compared.

Results: No statistically significant differences were found between the groups regarding age, hormone levels, antral follicle count (AFC), duration of COH, number of retrieved oocytes, M2 oocyte ratio, fertilization rate, or live birth rates (p>0.05).

Conclusion: Our findings suggest that both rFSH+rLH and rFSH+HMG combinations yielded similar clinical and laboratory outcomes in patients with diminished ovarian reserve. Given the observational nature of this study, these results should be interpreted with caution, and treatment protocols should remain individualized.

## Introduction

Success in assisted reproductive technologies (ART) is closely related to the acquisition of an adequate number of high-quality oocytes. However, some patients demonstrate a suboptimal response to standard controlled ovarian hyperstimulation (COH) protocols due to diminished ovarian reserve. This condition is defined as “poor ovarian response” (POR) and significantly limits the success of in vitro fertilization (IVF) procedures [1-3].

Managing patients with diminished ovarian reserve remains one of the most controversial areas in ART. The low quantity and quality of oocytes in these patients adversely affect embryo development and pregnancy outcomes [4,5]. Therefore, various stimulation protocols, gonadotropin combinations, and adjuvant treatment options have been investigated.

The main agents used in gonadotropin therapy include recombinant follicle-stimulating hormone (rFSH), human menopausal gonadotropin (HMG), and recombinant luteinizing hormone (rLH). HMG is a preparation that contains both FSH and LH activity. The rFSH+rLH combination is considered a more physiological approach and contributes to folliculogenesis via the two-cell-two-gonadotropin theory [6,7]. LH stimulates androgen production in theca cells, which is then aromatized into estrogen in granulosa cells, facilitating follicular development and oocyte maturation [8].

Some studies suggest that LH supplementation may improve outcomes, particularly in certain patient subgroups such as advanced maternal age, hypogonadotropic hypogonadism, or recurrent IVF failure [9]. However, it remains unclear whether this benefit extends to younger patients with diminished ovarian reserve [10]. Additionally, meta-analyses have reported inconsistent results regarding the routine use of LH supplementation [11,12].

Studies comparing HMG with the rFSH+rLH combination have generally demonstrated similar outcomes regarding embryo quality, fertilization rates, and clinical pregnancy rates [13,14]. However, variations may exist depending on individual patient characteristics. Consequently, a personalized treatment approach is increasingly emphasized in the selection of gonadotropin protocols [15].

This study aimed to compare the clinical, laboratory, and pregnancy outcomes of rFSH+rLH versus rFSH+HMG combination protocols in women with POR undergoing controlled ovarian stimulation with antagonist protocol, and to determine whether one regimen offers superior reproductive outcomes, focusing on the live birth rate as the primary measure of efficacy.

## Materials and methods

Study population and groups

This retrospective, single-center study included 90 patients diagnosed with POR who underwent COH and embryo transfer between 2023 and 2024 at the IVF unit of a tertiary unit hospital. All patients were followed up until delivery. Ethical approval for the study was obtained from the ethics committee of the local state university (approval number: 2024/453). The primary endpoint of this study was the live birth rate, defined as the delivery of at least one live newborn after 24 weeks of gestation. Secondary endpoints included the number of retrieved oocytes, fertilization rate, and clinical pregnancy rate.

From the data of 106 retrospectively screened patients, a total of 16 patients/cycles who underwent oocyte pickup (OPU) but did not meet the criteria for the primary analysis of the study were excluded from the analysis. These exclusions were considered cycle cancellations indicating inadequate response or laboratory failure in the POR population. Reasons for exclusion are as follows: cycles in which no oocytes could be obtained (No Oocyte): N = 4; cycles in which no mature (M2) oocytes could be obtained: N = 6; cycles with fertilization failure or pause in embryo development on day three: N = 6. After these exclusions, 90 patients who underwent OPU, obtained M2 oocytes and reached embryo development on day three constituted the final cohort of our study. When the files of the patients excluded from the study were examined, it was not deemed necessary to focus on this in detail, as the number of cancellations was equal in both groups and this was an expected limitation considering the physiology of POR patients.

Patients were defined as having POR according to the Bologna criteria [3]. The inclusion criteria were at least two of the followings: advanced maternal age (above 40 years) or other risk factors (endometriosis, previous ovarian surgery, history of chemotherapy/radiotherapy), a previous POR (three or less oocytes retrieved), abnormal ovarian reserve tests (e.g., anti-Müllerian hormone (AMH) level less than 1.1 ng/mL or antral follicle count (AFC) less than 5-7) [3].

Patients were assigned to the treatment protocol based on clinician decision and/or drug supply status at that time. Baseline prognostic factors were statistically compared to ensure no systematic bias was introduced during the allocation process. While the Bologna criteria were used for inclusion, the study cohort specifically represents Poseidon Groups 3 and 4 (patients with low ovarian reserve markers: AFC <5 and/or AMH <1.2 ng/mL) [16]. Retrospective review of the treatment protocol did not identify any systematic bias in this selection towards a particular subgroup of patients (e.g., assignment of patients with a better prognosis to one group). Protocol selection was based on the requirement that both agents (rFSH+rLH and rFSH+HMG) provide adequate LH activity alongside FSH activity.

Patients were divided into two groups based on the gonadotropin combination used in their stimulation protocol: the Pergoveris group (rFSH+rLH, n = 49) and the rFSH+HMG group (rFSH+HMG, n = 41).

All patients received the gonadotropin-releasing hormone (GnRH) antagonist protocol on days two to three of the menstrual cycle. Stimulation was initiated with a standardized high starting dose of 300 IU daily to maximize potential oocyte retrieval. Dosing was response-based rather than weight-based, and modifications were not routinely performed to maintain consistency. Patients meeting the criteria for POR were initiated on gonadotropins at a high starting dose of 300 IU daily (as a combination of rFSH+rLH or rFSH+HMG) to maximize potential oocyte retrieval. Follicular monitoring was performed via transvaginal ultrasonography (Voluson S6 Ultrasound System; GE Healthcare, Pfaffing, Austria). In Group 1, patients received 150 IU of rFSH (Gonal-f®; Merck-Serono, Istanbul, Turkey) plus one vial of Pergoveris® (150 IU rFSH + 75 IU rLH; Merck-Serono). In Group 2, patients received 150 IU of rFSH (Gonal-f®) plus 150 IU of highly purified HMG (HP-HMG) (Menopur®; Ferring, Maslak, Turkey). To prevent ovulation, a flexible antagonist protocol was used. GnRH antagonist (Cetrorelix acetate 0.25 mg, subcutaneous; Merck-Serono) administration was started on the day the diameter of the leading follicle reached 13-14 mm solely based on follicular diameter (flexible protocol) without additional hormonal threshold, and continued daily until the trigger (human chorionic gonadotropin (hCG)) day.

Ovulation triggering was performed with 250 µg of recombinant hCG (Ovitrelle®; Merck-Serono) plus 0.2 mg of triptorelin acetate (Gonapeptyl®; Ferring) when at least one leading follicle reached ≥18 mm in diameter. Oocyte retrieval was conducted 36 hours later. Retrieved oocytes were fertilized via intracytoplasmic sperm injection (ICSI).

D3 embryos were scored according to the following criteria: grade I-blastomere number (BL) of 6-10, of equal size, and fragmentation (FR) = 0%-5%; grade II-BL = 6-10, slightly equal in size, and FR = 5%-24%; grade III-BL = 6-10, unequal in size, and FR = 25%-49% or BL = 4-5 or >10; and grade IV-severely unequal-sized blastomeres, or FR > 50%, or embryo arrest. Grades I, II, and III indicate good-quality embryos, of which grades I and II are top-quality, and grade IV indicates low-quality embryos [17,18].

A maximum of two embryos were transferred on day three post-retrieval. All patients received standardized luteal phase support consisting of 800 mg/day vaginal progesterone soft capsule (Progesteron 200 mg) starting on the day following OPU and continuing until the 10th week of gestation or a negative pregnancy test. Serum β-hCG levels were measured on the 12th day post-transfer. Clinical pregnancy was defined as the detection of a gestational sac by transvaginal ultrasound three weeks after embryo transfer.

All patients included in the study were followed up through a prospective follow-up system in which pregnancy outcomes were followed until live birth. In addition to pregnancies being followed up within our hospital, pregnancy follow-up and birth information for patients coming from out of town were obtained by confirming with telephone calls and medical records. Through this prospective follow-up, live birth rates were confirmed as the primary clinical measure used to evaluate the effectiveness of protocols.

Statistical analysis

Descriptive statistics were presented as mean ± standard deviation or median (minimum-maximum) for numerical variables, and as frequency and percentage for categorical variables. For intergroup comparisons, the independent samples t-test was used for normally distributed numerical variables, and the Mann-Whitney U test was used when normality was not met. The chi-square test was applied for categorical variables. A p-value <0.05 was considered as statistically significant. Analyses were performed using IBM SPSS Statistics version 23.0 (IBM Corp., Armonk, NY, USA).

## Results

In our study, data from 90 patients who met the inclusion criteria were retrospectively analyzed. All patients had undergone OPU, had mature (M2) oocytes retrieved, achieved fertilization via ICSI, and reached day three embryo development. Among them, 49 patients were included in the group that received the rFSH+rLH combination (Group 1), while 41 patients were assigned to the group that received the rFSH+HMG combination (Group 2).

When comparing the two groups in terms of age, body mass index (BMI), hormonal profile, and AFC, no statistically significant differences were observed (Table 1). The mean age was 33.7 ± 5.5 years in Group 1 and 32.5 ± 4.6 years in Group 2 (p = 0.276). BMI values were 26.7 ± 5.3 and 26.1 ± 4.6, respectively (p = 0.594). Similarly, no significant differences were observed in FSH, AMH, or AFC levels between the groups (p > 0.05) (Table 1).

**Table 1:** Comparison of demographic and hormonal data of the groups BMI: body mass index, FSH: follicle-stimulating hormone, E2: estradiol, AMH: anti-Mullerian hormone, AFC: antral follicle count, rFSH: recombinant FSH, rLH: recombinant luteinizing hormone, HMG: human menopausal gonadotropin ¥: Independent sample t-test; §: Mann Whitney U Test; SD: Standard deviation.

	rFSH+rLH (n=49)	rFSH+HMG (n=41)		
Parameter	Mean±SD	Mean±SD	Test result	p
Age	33.7±5.5	32.5±4.6	1.096	0.276¥
Infertility duration	6.2±6.1	5.1±3.9	965.000	0.747§
BMI (kg/m²)	26.7±5.3	26.1±4.6	0.535	0.594¥
FSH	7.2±2.8	8±3.1	845.000	0.193§
LH	3.8±1.7	3.6±2	875.000	0.284§
E2	41.2±14.4	46.6±18.3	862.000	0.248§
AMH	0.7±0.3	0.6±0.3	894.000	0.369§
AFC	4.5±1.5	4±1.5	842.000	0.178§

The mean duration of COH was 9.7 days in both groups. The total dose of FSH used was 2909.2 ± 754.9 IU in Group 1 and 3183.5 ± 782.5IU in Group 2, with no statistically significant difference (p = 0.060). The total dose of LH used was 787.2 ± 258.2 IU in Group 1 and 1719.5 ± 644.6 IU in Group 2, with a statistically significant difference (p = <0.001). Endometrial thickness was similar between groups (p = 0.467). The mean number of retrieved oocytes was 4.1 ± 1.5 in Group 1 and 4.3 ± 1.2 in Group 2 (p = 0.316). The number of mature M2 oocytes was similarly distributed, with means of 3.0 ± 1.5 and 3.4 ± 1.4, respectively (p = 0.100). These findings indicate that both protocols resulted in comparable oocyte responses. The fertilization rate was 96.6% ± 21.5 in Group 1 and 94.2% ± 11.5 in Group 2 (p = 0.258), showing no statistically significant difference. Day three embryo quality was comparable between the groups, with no statistically significant difference observed. According to β-hCG measurements, 21 patients (42.9%) in Group 1 and 17 patients (41.5%) in Group 2 tested positive (p = 0.894). Clinical pregnancy rates were also similar between the groups, without statistical significance.

These findings suggest that rFSH+rLH and rFSH+HMG protocols exhibit comparable efficacy in patients with poor ovarian reserve in terms of oocyte response, embryo quality, and pregnancy rates (Table 2).

**Table 2:** Comparison of stimulation and laboratory data FSH: follicle-stimulating hormone, COH: controlled ovarian hyperstimulation, rFSH: recombinant FSH, rLH: recombinant luteinizing hormone, HMG: human menopausal gonadotropin ¥: Independent sample t-test; §: Mann Whitney U Test; SD: Standard deviation.

	rFSH+rLH (n=49)	rFSH+HMG (n=41)		
Parameters	Mean±SD	Mean±SD	Test result	p
COH duration (days)	9.7±1.5	9.7±1.4	0.022	0.982¥
Total FSH	2909.2±754.9	3183.5±782.5	773.500	0.060§
Total LH	787.2±258.2	1719.5±644.6	95.500	<0.001§*
E2 level on trigger day (pmol/L)	1632.4±800.9	1596.7±84.2	0.206	0.837¥
Endometrial thickness on trigger day (mm)	9.58±1.72	9.35±1.16	0.730	0.467¥
Number of oocytes	4.1±1.5	4.3±1.2	885.000	0.316§
Number of M2 oocytes	3±1.5	3.4±1.4	806.000	0.100§
Number of 2 pronuclei (PN)	2.9±1.5	3.2±1.3	847.500	0.194§
FertilRate (%)	96.6±21.5	94.2±11.5	911.000	0.258§
Number of top-quality embryos (day 3)	2.3±1.4	2.3±1.1	908.000	0.415§

Clinical outcomes and pregnancy rates

No statistically significant differences were observed between the two groups regarding infertility type (primary vs. secondary), treatment outcome (positive vs. negative), or pregnancy outcome (chemical pregnancy, miscarriage, live birth). The proportion of patients with primary infertility was 71.4% in Group 1 and 80.5% in Group 2 (p = 0.319). Positive pregnancy rates were 42.9% in Group 1 and 41.5% in Group 2 (p = 0.894). Live birth rates were comparable, reaching approximately 76% in both groups (Table 3).

**Table 3:** Clinical outcomes and pregnancy rates rFSH: recombinant follicle-stimulating hormone, rLH: recombinant luteinizing hormone, HMG: human menopausal gonadotropin χ² test

Variable	Group	rFSH+rLH n=49 (%)	rFSH+HMG n=41 (%)	Test result	p value
Infertility type	Primary	35 (71.4)	33 (80.5)		0.319
	Secondary	14 (28.6)	8 (19.5)	0.992	
Treatment Result	Positive	21 (42.9)	17 (41.5)	0.018	0.894
	Negative	28 (57.1)	24 (58.5)		
Pregnancy Outcome	Chemical Pregnancy	3 (14.3)	3 (17.6)	0.229	0.892
	Abortus	2 (9.5)	1 (5.9)		
	Live birth	16 (76.2)	13 (76.5)		

Chemical pregnancy and miscarriage rates were also similar, with no statistically significant differences between groups (chemical pregnancy: 14.3% vs. 17.6%; miscarriage: 9.5% vs. 5.9%; p = 0.892) (Table 3).

These results indicate that both treatment protocols provide comparable clinical success in patients with POR, and the live birth rates achieved in both groups are notably satisfactory.

## Discussion

POR remains one of the most critical limiting factors in ART. Defined by the Bologna criteria, this population is characterized by both a reduced number and quality of oocytes, which negatively affects embryo development and pregnancy success rates. To date, no consensus has been reached regarding the optimal stimulation protocol for these patients [4].

In this study, we compared the clinical and laboratory outcomes of rFSH+rLH and rFSH+HMG combination protocols in patients with POR. Our findings revealed no statistically significant differences between the two groups in terms of the number of retrieved oocytes, proportion of M2 oocytes, fertilization rate, embryo quality, or pregnancy outcomes. However, several methodological issues must be acknowledged. First, the retrospective and non-randomized design introduces the potential for treatment allocation bias. Although baseline characteristics were statistically comparable, the observational nature limits our ability to draw definitive causal inferences.

Similarly, a randomized controlled trial by Tehraninejad et al. also reported no significant difference in pregnancy rates between rFSH+rLH and rFSH+HMG protocols [19]. Supporting the differences in sources of LH bioactivity, Kirshenbaum et al. found that stimulation with rFSH+rLH resulted in a significantly higher number of mature and fertilized oocytes compared to HP-HMG; however, pregnancy rates remained statistically comparable [20]. These findings suggest that both protocols exhibit similar effectiveness in patients with POR.

Our findings indicate that the use of HMG yields comparable results to the rFSH+rLH combination. This aligns with previous randomized controlled trials, such as Pacchiarotti A et al., which compared these two stimulation protocols in IVF cycles and reported similar outcomes in terms of embryo quality, fertilization rates, and clinical pregnancy rates [14].

The effect of LH supplementation on folliculogenesis and oocyte maturation remains a subject of debate. Kolibianakis et al., in their systematic review, suggested that endogenous LH levels may significantly influence pregnancy probability during IVF cycles [21]. This implies that LH supplementation should be carefully evaluated, particularly in patients with low endogenous LH levels. LH receptors are expressed not only in granulosa cells but also in theca and stromal cells, playing a regulatory role in steroidogenesis [6]. LH stimulates androgen production in theca cells, which are then aromatized into estrogens by granulosa cells, creating the hormonal environment essential for follicular development and oocyte maturation [8]. LH deficiency, especially in women of advanced age or those with hypogonadotropic hypogonadism, may negatively affect oocyte quality. Bosch et al. indicated that LH supplementation could improve fertilization and embryo quality [9]. However, whether this benefit extends to younger patients with diminished ovarian reserve remains unclear [10].

Andersen et al. reported that administration of LH during the late follicular phase enhanced embryo quality and pregnancy rates [7]. Nevertheless, meta-analyses have not provided strong evidence supporting the universal benefit of LH supplementation [11,12]. Similarly, Mochtar et al. noted that rLH supplementation may improve oocyte quality and implantation potential in selected subgroups, but does not consistently benefit the general IVF population.

HMG contains both FSH and LH-like activity, which may be sufficient for this patient population without the need for additional LH supplementation [14,15]. Moreover, some studies have evaluated HMG as a more cost-effective alternative to rFSH+rLH, yielding comparable pregnancy outcomes [22].

Our results suggest that LH supplementation may only be beneficial in selected patient populations. Specifically, its role may be more relevant in cases involving advanced maternal age, low endogenous LH levels, or hypogonadotropic conditions [23]. However, its value in young, normogonadotropic patients remains questionable, as some studies have shown no clear benefit in terms of pregnancy outcomes despite increased oocyte yield [24].

One of the major strengths of this study is the inclusion of a homogeneous patient population diagnosed with POR according to the Bologna criteria. A standardized antagonist protocol was applied uniformly, and gonadotropin dosing was consistent across both treatment groups. The single-center design ensured standardized clinical practices and embryo assessments. Moreover, the direct comparison between two commonly used gonadotropin regimens, rFSH+rLH and rFSH+HMG, enhances the clinical applicability of the findings. The comprehensive evaluation of clinical, laboratory, and pregnancy outcomes, including live birth rates, adds further strength to the study.

The exclusion rates are an expected limitation given the physiology of POR patients. Our study compared the clinical efficacy of two protocols containing different LH activities, specifically in patients who completed stimulation and reached the stage suitable for embryo transfer. Future studies could prospectively examine the effects of these two protocols on cycle cancellation rates.

In our study, the two-fold difference in LH between the two groups and the fact that both protocols provided similar oocyte yield, embryo quality, and live birth rates despite this dose difference suggest that LH activity above a certain threshold may be sufficient for POR patients and that a two-fold dose increase does not provide any additional clinical benefit. This finding makes an important contribution to the debate about the optimal dose of LH supplementation.

The retrospective nature of our study lacks the power to make definitive causal inference provided by randomized controlled trials. The relatively small sample size may reduce statistical power to detect differences in some variables. The absence of genetic testing (preimplantation genetic testing for aneuploidies (PGT-A)) of embryos restricts the ability to assess embryonic competence in depth. However, this single-center study was conducted by the same clinician/center from all drug selection to completion of treatment. This minimizes inter-center and inter-clinician heterogeneity and the risk of biased decisions based on specific data or parameters of practice. Furthermore, retrospective records better reflect clinical practice conditions, supporting the absence of commercial bias or deliberate patient selection in protocol selection. Therefore, our findings provide valuable evidence of the inherent clinical efficacy we see in daily clinical practice.

## Conclusions

In conclusion, our data suggest that LH supplementation may offer benefits in selected subgroups, but a personalized approach remains essential for patients with POR. While these protocols appear equally effective in this cohort, the findings should be interpreted with caution due to the study's observational nature. These results should ideally be validated by larger-scale prospective randomized controlled trials. Based on the totality of the evidence, the choice of gonadotropin combination should be tailored to the individual, taking into account factors such as patient age, hormonal profile, previous ovarian response, and financial considerations.
